# Visual attentional differences in psychology students with and without disabilities: a pilot study assessing the flanker task for prescriptive visual accommodative technologies

**DOI:** 10.3389/fpsyg.2025.1484536

**Published:** 2025-03-27

**Authors:** Anders Chan, Zachary I. Harkinish-Murray, Sabrina Colmone, Jessica E. Orens, Sharon Thomas, Nicole Albanese, Katherine McCabe, Rui Freitas, Stephanie P. Bailey, Ravi L. Ramdhari, Michael T. Verrengia, Kainaat F. Siddiqui, Oscar E. Lopez, Stacey DeFelice, Basabi Runi Mukherji, Lorenz S. Neuwirth

**Affiliations:** ^1^Department of Psychology, SUNY Old Westbury, Old Westbury, NY, United States; ^2^SUNY Neuroscience Research Institute, SUNY Old Westbury, Old Westbury, NY, United States; ^3^Department of Biological Sciences, SUNY Old Westbury, Old Westbury, NY, United States; ^4^Office of Services for Students with Disabilities, SUNY Old Westbury, Old Westbury, NY, United States

**Keywords:** visual eye tracking, Gazepoint eye tracking, visual distractions, visual accommodative technologies, undergraduate psychology students, students with a disability, visual attention, pilot study

## Abstract

**Introduction:**

The percentage of college students with disabilities has been growing and has doubled in the last two decades; thus, students with disabilities are pursuing college degrees in increasing numbers. Unfortunately, this population growth has not been matched with growth in available accommodative technologies in institutions of higher learning. Colleges and universities often do not have resources to fund and provide specific accommodative technology and support for this steadily increasing population. What is worse is that there is also a lag in emergent assessment and screening tools which are required to match student disabilities with appropriate accommodative technologies, resulting in a mismatch between student needs with *appropriate* accommodative technologies. The present pilot study was conducted with students with a range of disabilities, such as learning disabilities, emotional or psychiatric conditions, orthopedic or mobility impairments, attention-deficit disorder/attention-deficit hyperactivity disorder, health impairments (HI), and multiple disabilities, which were assessed using a Flanker Task, specifically to determine how sensitive it was in detecting differences in their visual attention performance. This information could be used to predict whether the student would benefit from specific accommodative technologies.

**Materials and methods:**

Undergraduate psychology students with and without disabilities volunteered to participate in a triple-blind study that sought to investigate whether their visual attention performance on a 10-min Flanker Task could be used to predict which students might benefit from visual accommodative technologies. The first experiment was used as a negative control to assess whether environmental distractions could interfere with participant visual attention. The second experiment compared the Flanker Task performance of students with and without disabilities in a controlled Neuropsychology Laboratory sound-attenuated environment. The third experiment evaluated the cumulative records for percent (%) accuracy and reaction times (RTs) for students with and without disabilities to examine patterns in visual attentional performance. The fourth experiment disaggregated the students with disabilities and examined their patterns in visual attentional performance.

**Results:**

The results showed the Flanker Task was sensitive in detecting differences in students’ visual attention performance between noisy and controlled environments differentiated students with and without disabilities. Furthermore, when students with disabilities were aggregated, their Flanker Task cumulative records were sensitive in detecting shifts in their visual attention behavior patterns. Lastly, the Flanker Task cumulative records were also sensitive in detecting disaggregated students with disability differences in their visual attention performance.

**Conclusion:**

The pilot study proved promising that a 10-min Flanker Task can be used as an effective screening tool to match students with disabilities with appropriate accommodative technologies based on their visual attentional abilities. This type of screening tool is easy to create, has minimal cost, and can be implemented quickly. This provides colleges and universities with an easy approach to assessing the needs of students with disabilities and tailoring appropriate assistive technologies.

## 1 Introduction

The percentage of college students with disabilities has been growing and has doubled in the last two decades, increasing from 11% in 2004 to 21% in 2020 (U.S. Government Accountability Office, 2024), and this growth does not seem to have slowed to date. U.S. Government Accountability Office (2024) report also indicated that students with disabilities are pursuing college degrees in increasing numbers as compared to past decades, making it incumbent for colleges and universities to increase their efforts and commit more resources to mitigate the challenges faced by this growing population of students. However, these institutions of higher learning may not be able to provide all the necessary resources and services that students with disabilities may need to optimally succeed, and what is worse is that there is also a lag in emergent assessment and screening tools which are required to match student disabilities with appropriate accommodative technologies, resulting in a mismatch between student needs and available and *appropriate* accommodative technologies (Grigal et al., 2022; Kim and Kutscher, 2021; Newman et al., 2021; Becht et al., 2020; Carroll et al., 2020; Fleming et al., 2017).

The ability to service the enrollment growth in the population of students with disabilities has been complicated by another factor: COVID-19. New York was the first hot spot of contagion of the coronavirus-19 (COVID-19) within the US, in March of 2020. This resulted in an educational context that required transitioning from in-person classes to online learning with extraordinary rapidity. There were a variety of online learning environments: synchronous (i.e., remote learning with an instructor actively engaging students virtually) or asynchronous (i.e., a passive online only experience with no instructor presence) formats. The rapid change in these formats negatively impacted students learning across the board and disproportionately affected students with disabilities (Gin et al., 2002; Gin et al., 2021; Meleo-Erwin et al., 2021; Neuwirth et al., 2021a,b). Moreover, the associated negative impacts of COVID-19 on students’ social and physical contact as well as on their ongoing social development contributed to a rise in mental health concerns and the need for matched urgent response efforts to best address undergraduate student needs (Wood et al., 2024; Salimi et al., 2023; Conrad et al., 2021; Lee et al., 2021; Li et al., 2021; Neuwirth et al., 2021a,b; Patias et al., 2021; Chirikov et al., 2020). U.S. Government Accountability Office (2024) report indicated one of the factors that has fueled the growth of students with disabilities is that of increasing numbers of students being diagnosed with mental health conditions. Notably, the pandemic exacerbated that trend.

Student accessibility to a range of curricular activities and feeling a sense of inclusion in such educational practices was reported to mitigate against these effects for students with disabilities at well-funded undergraduate institutions of higher learning within the US (Campanile et al., 2022). But what were the implications for less well funded institutions of higher learning? How did students at Primarily Undergraduate Institutions (PUIs), Minority Serving Institutions (MSIs), Hispanic Serving Institutions (HSIs), and Asian American and Pacific Islander Serving Institutions (AAPISIs), which are typically less well-funded, fare? How do these less funded colleges and universities student experiences differ from that of colleges and universities in New York post-pandemic? This is a critical question in which careful examination of several factors is needed to best understand not just what educational hardships students with and without disabilities experienced, but rather what lessons were learned. We need to continually examine and reassess which strategies were effective in providing the best learning environments since classes have become more likely to be hybrid, remote, or online, post-pandemic.

Self-reported psychological stress in students at colleges and universities had already been trending upward from 2010 to 2018. The National Center for Education Statistics (NCES) data from 2011 and 2012 reported that 11% of undergraduate students identified as having a disability with nearly 38% of students with disabilities were enrolled in 2-year as compared to 9.8% at 4-year institutions (U.S. Department of Education, National Center for Education Statistics, 2015). These students with disabilities academic performance were also likely to have been exacerbated by COVID-19 (Knapstad et al., 2021). In addition, students with and without disabilities required assistive and accommodative technologies to ensure access to, compliance with, and optimizing student inclusivity when dealing with the unprecedented learning circumstances brought on by the COVID-19 pandemic (McNicholl et al., 2021; Newman et al., 2021; Gin et al., 2020; Mamboleo et al., 2020; Moriña et al., 2020; Zhang et al., 2020). It is also important to note that at varying educational levels, teacher burnout and turnover were more likely to occur when teaching students with disabilities (Gilmour and Wehby, 2020). Taken together, there is a very complex set of factors that contribute to undergraduate college students’ psychological wellbeing that require more systematic and careful exploration (Morales-Rodriguez et al., 2020). Despite 50 years of science and educational practices in understanding, teaching, and supporting students with disabilities, the field has less experience in teaching adults with very different learning needs today than in previous decades (Grigorenko et al., 2020). Interestingly, with the advancement of neuropsychology research technology, visual eye tracking has been shown to be effective in differentiating college student learning in students without disabilities (González-Diez et al., 2023; Lee, 2023) as well across sub-groups of students with disabilities, some assessed from childhood through adulthood: autism (Tang, 2022; Banire et al., 2020; Mo et al., 2019); neurodevelopmental disorders (Bilbao et al., 2024); neurodivergent classrooms (Wong et al., 2023); learning disabilities (Liu et al., 2024; Rizwana, 2019); reading disabilities (Solan et al., 2001); dyslexia (Caldani et al., 2020; Kim and Wiseheart, 2017; Kim et al., 2016; Kim et al., 2014); attention-deficit/hyperactivity disorders (AD/HD) (Caldani et al., 2022), schizophrenia (Wang et al., 2022; Qin et al., 2022; Navalón et al., 2021; Huang et al., 2020); and even the impacts of COVID-19-related psychosis (Zhang et al., 2022), to name a few. Furthermore, there are ample data on the use of different visual accommodative software technologies to help students with disabilities (e.g., Kurzweil 3000, Livescribe, and AccessText). The evidence for assessment and screening data that prognostically match students with specific disabilities to these accommodative technologies to best serve them is lacking. There are, however, some emerging efforts to address this matter (see Lev et al., 2022; Hewett et al., 2020; Bacon et al., 2020; Dahmani et al., 2020; Kröger et al., 2020; Yaneva et al., 2020).

There is a paucity of research investigating (1) the cognitive processing in people with multiple disabilities; (2) the availability of tools that differentiate learners with multiple disabilities; (3) the *efficacy* of technologies provided to students with disabilities; and (4) the educational outcomes when using such technologies. Scant data exist on whether assistive or accommodative technologies are sensitive enough to accommodate individual needs or are tailored to support people with specific or multiple disabilities. There are several reports on college students examining their visual attention in cases of attention-deficit/hyperactivity disorder (Liebel and Nelson, 2017), executive functioning concerns (Grieve et al., 2014), how distracting conditions impair visual–spatial working memory (Lineweaver et al., 2012), etc. whereby more reports are isolated to children and young adolescents that are not college-age. This presents a gap in the literature from the times in which children with disabilities may encounter more early intervention opportunities and supports for their disabilities that might be either less available or offered in college-specific and/or college-dependent ways when these children enroll in college. To deconstruct these problems, the present study sought to group undergraduate college students with disabilities into six main categories: learning disabilities (LD), emotional or psychiatric conditions (EPC), orthopedic or mobility impairments (OMI), attention-deficit/hyperactivity disorder (AD/HD), health impairments (HI), and multiple disabilities (MD). The aim of this study was to determine whether assessing student’s visual attention processing abilities through a 10-min Eriksen Flanker Task (Eriksen and Eriksen, 1974) could be used as a prognostic tool to screen and differentiate students with disabilities based on the six main disability sub-groups, as described above. It was hypothesized that the eye gaze software technology with the Flanker Task could detect visual attentional differences based upon the type of disability between a triple-blinded student with and without a disability. In addition, it was hypothesized that the Flanker Task could be used as a prognostic tool to screen students with specific disabilities through detecting differences in their eye tracking pattern data through the measures analyzed from the Flanker Task.

## 2 Methods

### 2.1 Participants

The present study was funded by a Faculty Development Grant (FDG) intended to pilot improving curricular development for teaching students with disabilities by determining which accommodative technologies would help a student to learn best given their unique disability. The pilot study was conducted on a subset of *N* = 155 4-year undergraduate college students (*n* = 111 students without disabilities and *n* = 44 students with disabilities) that were mostly majoring in Psychology. The study was approved by The State University of New York (SUNY) Old Westbury Institutional Review Board (IRB) and the SUNY Old Westbury Office of Services for Students with Disabilities (OSSD). The *n* = 44 students with disabilities consisted of learning disabilities, emotional or psychiatric conditions, orthopedic or mobility impairments, attention-deficit disorder/attention-deficit hyperactivity disorder (ADD/AD/HD), health impairments (HI), and multiple disabilities which included students with more than one psychiatric condition and requiring both different and high support needs. The Director of the OSSD was the only individual privy to the student’s disability status as they coordinate their services on campus. As students volunteered for the study, they were given coded numbers to conceal their disability status and were triple-blinded to the researchers running the actual Flanker Task. When students signed up to take the Flanker Task, they presented their code to the researchers, and this was used as the only identifier in the data. At the end of the study, the OSSD Director grouped the data identifiers and recoded them to then be sent to the researchers for further analyses. These unique codes could not be traced back to the students and were only known by the OSSD Director to ensure the confidentiality of the disability status of all participants. Both students with and without disability status were coded in this same manner. The students were then assessed using a Flanker Task, specifically to determine how sensitive it was in detecting differences in their visual attention performance. This information could be used to predict whether the student would benefit from specific accommodative technologies.

The participants consented to voluntarily take part in the pilot study to assess whether their visual attention performance could be both sensitive and predictive of the kinds of accommodative technologies that would best match their learning needs for their specific disability. The participants volunteered and were randomly assigned to participate in the pilot study across a set of four experiments. All participants were triple-blinded from the main investigators to ensure anonymity and unbiased analyses from the study’s findings.

### 2.2 Design and Procedures

**Figure 1:**
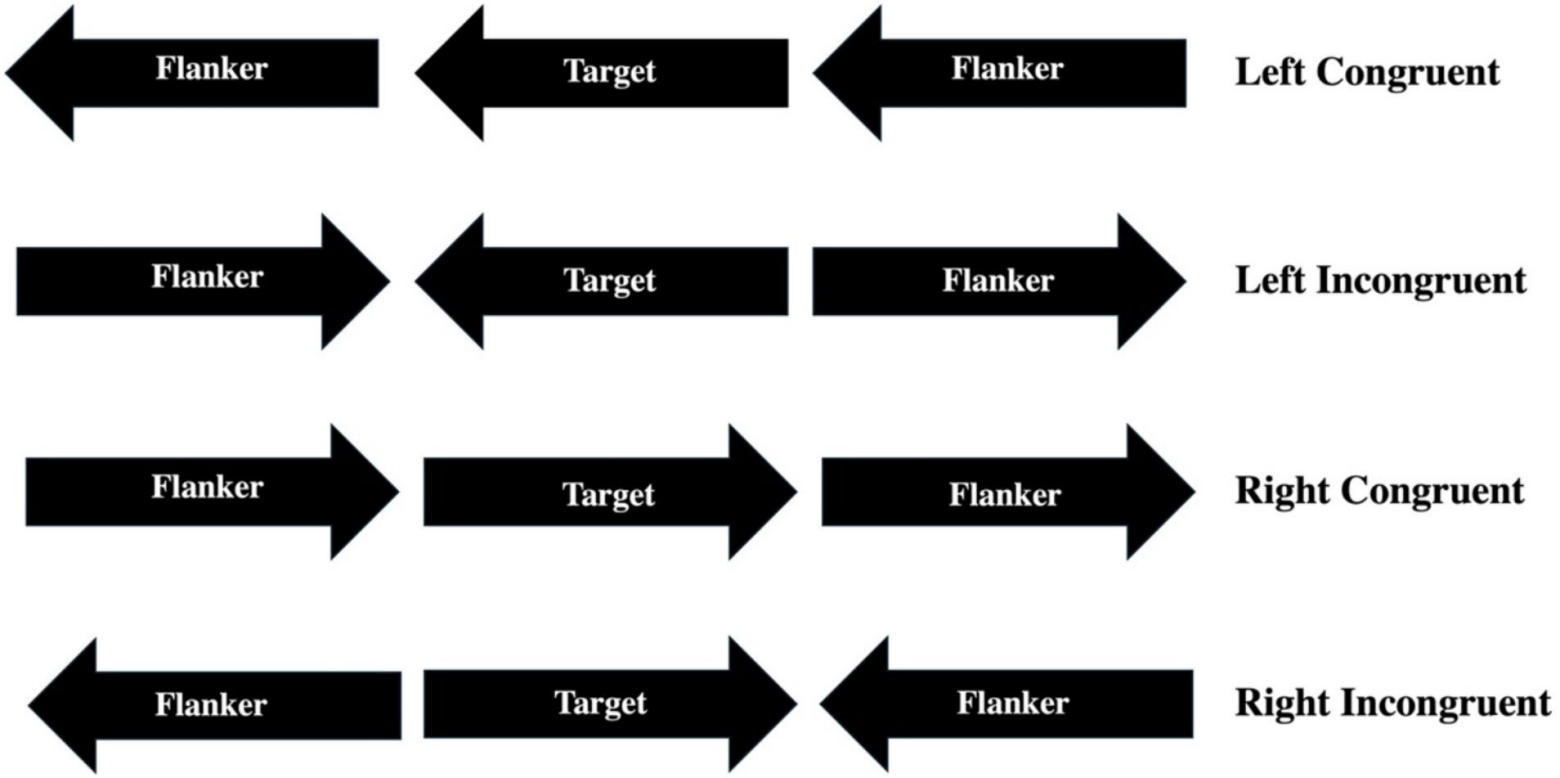
Eriksen Flanker Task stimuli that were presented to the participants in a random design with 10 trials for each test condition. The control trials were presented with just the center target arrow pointing left for 5 trials and right for 5 trials (not shown). Otherwise, the other test conditions were displayed as left congruent (top row), left incongruent (second row), right congruent (third row), and right incongruent (bottom row).

Participants were subjected to a classic Eriksen Flanker Task (Eriksen and Eriksen, 1974) that was adapted for selectivity and for investigating executive control (Lehle and Hübner, 2008; Stins et al., 2007; Kopp et al., 1996), and it was time locked with an eye gaze visual tracking system Gazepoint GP3 (Vancouver, BC) with the flanker stimuli (i.e., horizontal black arrows) presented visually on screen through Neurobehavioral Systems Inc. (Albany, CA). In brief, the flanker task was designed with a set of three arrows in which the target arrow remained in the middle of the screen and two flanker arrows were displayed to the left and right of the target arrow. The two flanker arrows were displayed in either congruent (i.e., facing the same direction as the target arrow) or incongruent (i.e., facing opposite directions when compared to the target arrow; see Figure 1). Participants were tasked with behaviorally selecting key responses to indicate either a congruent or incongruent flanker for each trial. Participants were given 12 practice trials before starting the experiment to familiarize themselves with the task. After the practice trails, participants entered the experiment which contained 50 trials that were randomized across 5 test conditions (i.e., 10 trials of Control, Left Congruent, Left Incongruent, Right Congruent, and Right Congruent arrows). The experiment lasted approximately 10–15 min. Participants *percent (%) accuracy* of correctly chosen trials across all experimental conditions, their *reaction time (RT)* measured in microseconds (μsec), and their *cumulative records* for each experimental condition for both *% accuracy* and *RT* were used to infer their cognitive attentional processes. In addition, their eye tracking data were used to determine how their visual gaze as a function of external environmental distraction (i.e., noise), disability (i.e., when aggregated together), and specific type of disability (i.e., when disaggregated) would suggest which type of visual accommodative technology might best match a student with a disability to use as educational learning resources while in college.

*Experiment 1:* A subset of (*N* = 61) participants were randomly assigned to test the sensitivity of the Flanker Task in distracting conditions between a Control Environment and a Noisy Environment. A total of *N* = 11 males (i.e., *n* = 4 Control Environment and *n* = 7 Noisy Environment) and *N* = 50 females (i.e., *n* = 26 Control Environment and *n* = 24 Noisy Environment) were examined on their *% accuracy* and *RT*. This experiment was used as an internal negative control to ensure that the eye gaze technology was sensitive to external environmental factors, before assuming that it could be sensitive to internal human factors (i.e., disability).

*Experiment 2:* A second subset of (*N* = 40) participants were randomly assigned to test the sensitivity of the Flanker Task to detect any differences between students with and without disabilities. A total of *N* = 6 males (i.e., *n* = 4 students without disabilities and *n* = 2 students with disabilities) and *N* = 34 females (i.e., *n* = 26 students without disabilities and *n* = 8 students with disabilities) were examined on their *% accuracy* and *RT*.

*Experiment 3:* A third subset of (*N* = 40) participants were randomly assigned to test the sensitivity of the Flanker Task to detect any differences between students with and without disabilities. A total of *N* = 20 males (i.e., *n* = 10 students without disabilities and *n* = 10 students with disabilities) and *N* = 20 females (i.e., *n* = 10 students without disabilities and *n* = 10 students with disabilities) were examined on their *% accuracy*, *RT,* and *cumulative records*.

*Experiment 4:* A third subset of (*N* = 14) participants were randomly assigned to test the sensitivity of the Flanker Task to detect any differences between students with different types of disabilities. A total of *N* = 14 participants with disabilities (i.e., *n* = 3 identified as having ADD/AD/HD, *n* = 3 identified as having a learning/processing disorder, *n* = 1 identified as having an emotional/psychiatric disorder, *n* = 2 identified as having an occupation/motor impairment, *n* = 3 identified as having a neurological/health impairment, and *n* = 2 identified as having multiple disabilities) were examined on their *% accuracy*, *RT,* and *cumulative records*.

### 2.3 Materials

**Figure 2:**
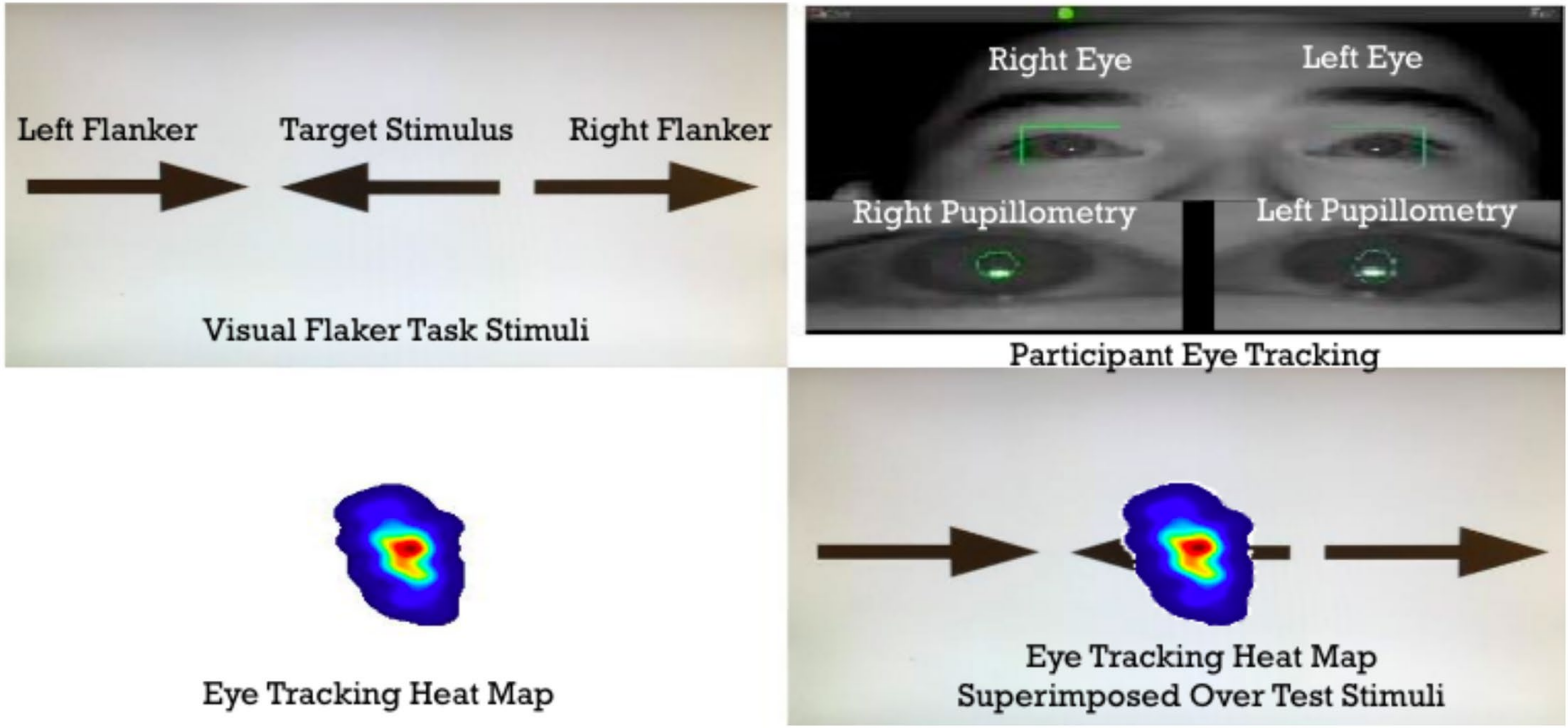
Flanker Task visual stimuli from the point of view of the participant (upper left panel), the Gazepoint eye gaze capture system detecting both left and right pupillometry (upper right panel), the combined fixation point of the left and right pupillometry forming an eye tracking heat map (hot colors = increased activity and cool colors = decreased activity; lower left panel), and the superimposed eye tracking heat map over the flanker task to detect deviations in the heat maps for each participant (lower right panel).

The participants signed a consent to volunteer to participate in the study. Participants were given a unique letter and number (e.g., A1 and B1) to be used as their data coding mechanism for their study and were triple-blinded throughout the study and even in their results. The participants were shown the Eriksen Flanker Task on a Dell laptop computer that was either in an uncontrolled noisy environment (i.e., a table in the main hallway of the university campus center that has the most foot traffic) or in the Psychology Department’s Neuropsychology Laboratory in a controlled sound-attenuated environment. The flanker stimuli (see Figure 1) were programmed using MATLAB (The MathWorks Inc., Natick, MA) and presented visually on screen through Neurobehavioral Systems Inc. (Albany, CA). The stimuli were programmed to display 12 practice trials before starting the experiment to familiarize participants with the task. After the practice trails, participants entered the experiment which contained 50 trials that were randomized across 5 test conditions (i.e., 10 trials of Control [target arrow presented alone either facing left or right], Left Congruent, Left Incongruent, Right Congruent, and Right Congruent arrows). The experiment lasted approximately 10–15 min. The Dell laptop computer was time locked with an eye gaze visual tracking system Gazepoint GP3 (Vancouver, BC) to track and map where the participant eyes were focusing during their response time for each of the trials prior to making a selection (Figure 2). Once the experiment ended, the participants were thanked for their participation and left the controlled or uncontrolled environment. Data were then downloaded from the computer into Microsoft Excel, reorganized, data visualizations graphed, and subsequently the data were transferred into SPSS version 24 (IBM^®^: Armonk, New York, United States) for later statistical analyses.

### 2.4 Statistical analyses

As this was a pilot study to evaluate the potential to use and incorporate more visual attention screening tools to determine which students would benefit most or have the most difficulty with visual accommodative technologies offered to them to support their undergraduate learning in the field of psychology, the statistical analyses solely focused on the *% accuracy* and *RT* as the dependent variables of the participants visual attention on the Eriksen Flanker Task (Eriksen and Eriksen, 1974). Since the disability group was aggregated and their combined visual eye tracking heat map did not differ from the no disability group (data not shown), it did not add to the study findings and was therefore excluded. Furthermore, for the disaggregated disability sub-group datasets, since the samples sizes were small and not enough to provide a normalized/representative group average visual eye tracking heat map, these data were also excluded. Instead, the disability sub-group data were evaluated using a single-subjects design for cumulative records to illustrate their *% accuracy* and *RT* over the 10 trials for each Flanker Task test condition after pseudo-randomization. The statistics were conducted using a general linear model univariate *analysis of variance (ANOVA)* between-subjects design followed by a *Tukey’s B honestly significant difference (HSD) test* for *pair-wise comparison* along with a *partial eta-square* (*η_p_*^2^) for determining *effect sizes* where applicable. The multi-factorial *ANOVA* consisted of *Environmental Test Condition* (Control vs. Noisy), *Disability* (no disability vs. disability), and *Flanker Test Condition* (Left Congruent, Left Incongruent, Right Congruent, and Right Incongruent) resulting in a 2X2X3 factorial design to assess the *main effects* and *interactions* between groups. This was followed by a *partial eta-square* for determining the *Effect Size* when appropriate to evaluate differences in *Environmental Test Condition*, *Gender*, and *Disability* as independent/quasi-independent variables between groups. All statistical analyses were conducted using SPSS version 24 (IBM^®^: Armonk, New York, United States). The criteria for statistical significance were set at *α* ≤ 0.05 with a *confidence interval* of 95% (*CI* = 95%). Data are presented visually as bar graphs ± *standard error of the means* (SEM) for the *% accuracy* and *RT*, whereas the cumulative records for the *% accuracy* and *RT* are presented visually as line graphs ± SEM.

## 3 Results

### 3.1 Experiment 1 findings

**Figure 3:**
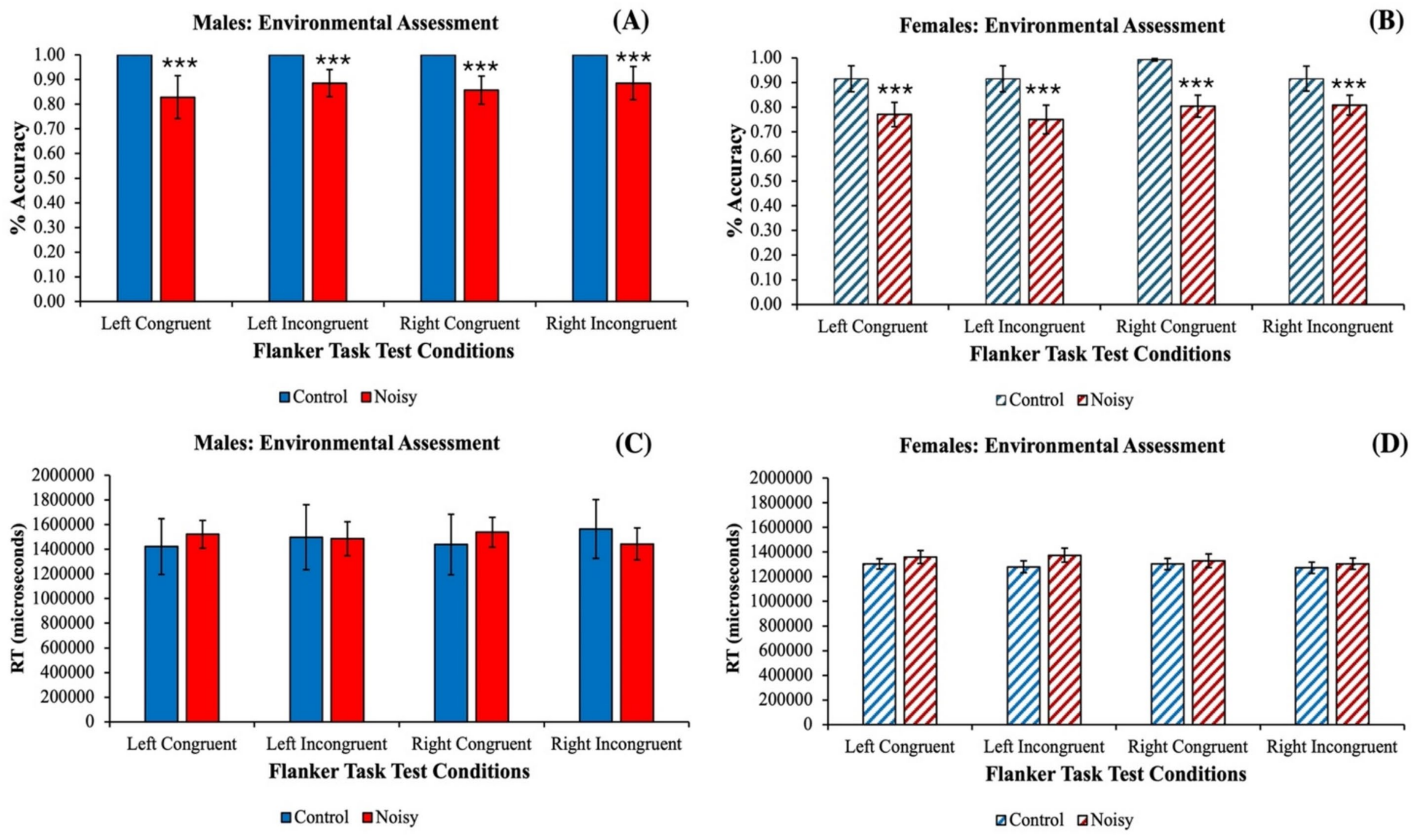
Differences between males (solid bars) and females (striped bars) for the % accuracy measure **(A,B)** and the differences between males (solid bars) and females (striped bars) for the reaction time (RT) measures in (μsec; **C,D**) as a function of the environment in which they were tested. The data show a statistically significant effect of a noisy environment that reduced the % accuracy in males (*p* < 0.001***; **A**) and females (*p* < 0.001***; **B**) across all Flanker Test Conditions. Interestingly, neither males **(C)** nor females **(D)** exhibited any statistically significant effect of a noisy environment that influenced RT across any of the Flanker Test Conditions. Data are shown as the Mean ± SEM, and bars without SEMs are because the value had little to no variability.

The results for males showed that the sensitivity of the eye gaze technology in distracting conditions between a Control Environment and a Noisy Environment for the *% accuracy measure* revealed a significant effect of *Environment* [*F*_(1)_ = 8.784, *p* = 0.005**, *η_p_*^2^ = 0.196]. However, there were no significant effects of *Flanker Test Condition* [*F*_(3)_ = 0.089, *p* = 0.966 n/s], nor was there any significant *Environment X Flanker Test Condition interactions* [*F*_(1,3)_ = 0.089, *p* = 0.966 n/s] for the *% accuracy measure* (Figure 3A). The results for females showed that the sensitivity of the eye gaze technology in distracting conditions between a Control Environment and a Noisy Environment for the *% accuracy measure* revealed a significant effect of *Environment* [*F*_(1)_ = 20.660, *p* = 0.001***, *η_p_*^2^ = 0.097] with a significant *pair-wise comparison* between the groups on the Left Congruent *Flanker Test Condition* (*p* = 0.031^#^), the Left Incongruent *Flanker Test Condition* (*p* = 0.014^##^), and the Right Congruent *Flanker Test Condition* (*p* = 0.005^##^). However, there were no significant effect of *Flanker Test Condition* [*F*_(3)_ = 0.750, *p* = 0.524 n/s], nor was there any significant *Environment X Flanker Test Condition interaction* [*F*_(1, 3)_ = 0.268, *p* = 0.849 n/s] for the *% accuracy measure* (Figure 3B). The results for males showed that the sensitivity of the eye gaze technology in distracting conditions between a Control Environment and a Noisy Environment for the *RT measure* revealed no significant effect of *Environment* [*F*_(1)_ = 0.018, *p* = 0.895 n/s], no significant effect of *Flanker Test Condition* [*F*_(3)_ = 0.012, *p* = 0.998 n/s], nor was there any significant *Environment X Flanker Test Condition interaction* [*F*_(1, 3)_ = 0.187, *p* = 0.904 n/s] (Figure 3C). The results for females showed that the sensitivity of the eye gaze technology in distracting conditions between a Control Environment and a Noisy Environment for the *RT measure* revealed no significant effect of *Environment* [*F*_(1)_ = 2.140, *p* = 0.145 n/s], no significant effect of *Flanker Test Condition* [*F*_(3)_ = 0.290, *p* = 0.832 n/s], nor was there any significant *Environment X Flanker Test Condition interaction* [*F*_(1, 3)_ = 0.192 *p* = 0.902 n/s] (Figure 3D). Overall, when examining the *Main Effect* of *Gender,* it was observed to be statistically significant for *% accuracy* [*F*_(1)_ = 3.626, *p* < 0.05*, *η_p_*^2^ = 0.097], and a *Main Effect* of *Environment* was also observed to be statistically significant for *RT* [*F*_(1)_ = 13.162, *p* < 0.001***, *η_p_*^2^ = 0.055].

### 3.2 Experiment 2 findings

**Figure 4:**
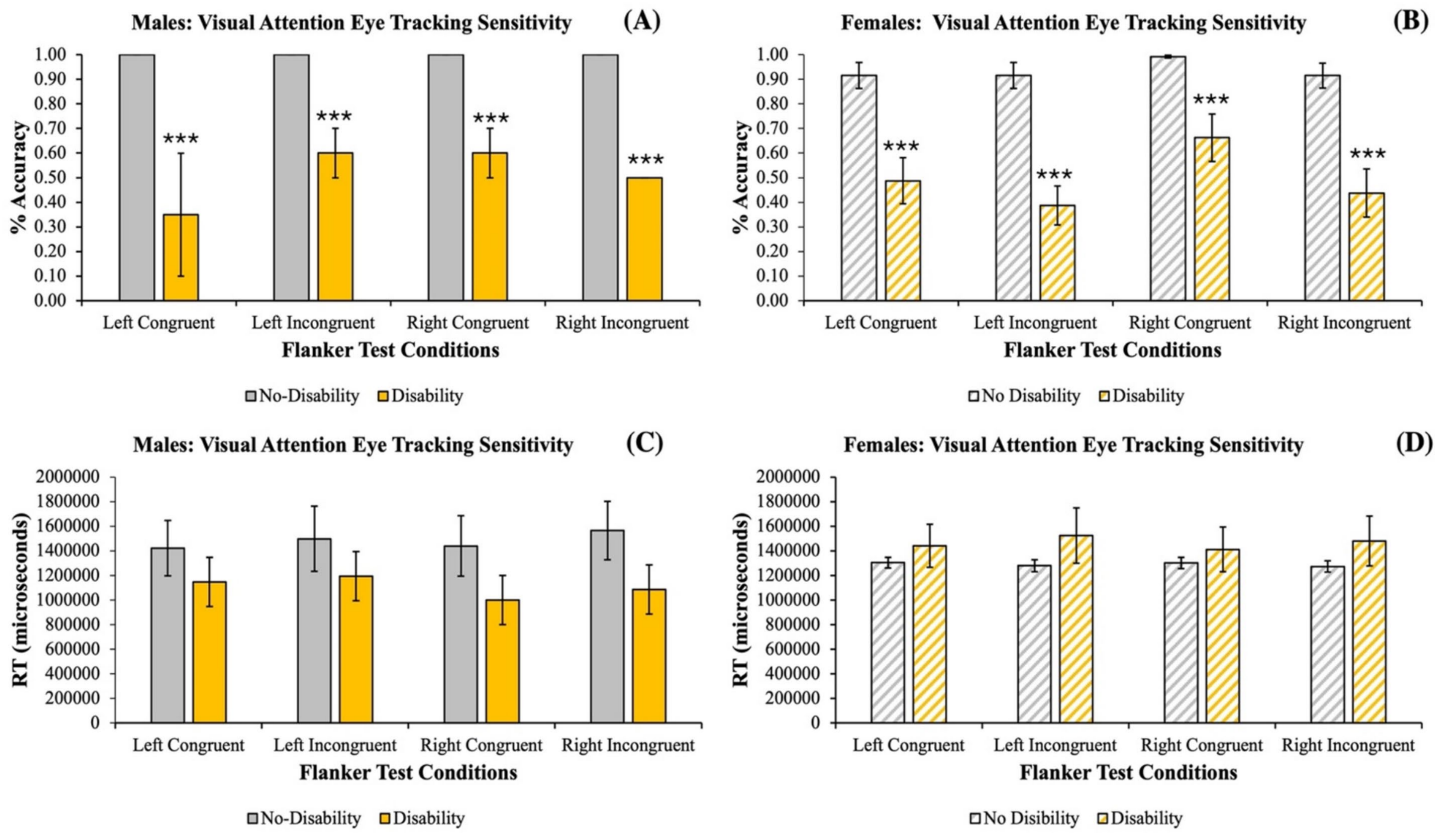
Differences between male participants with no disability (gray solid bars) and with a disability (yellow solid bars) and female participants with no disability (gray striped bars) and with a disability (yellow striped bars) for the % accuracy measure **(A,B)**. In addition, the differences between male participants with no disability (gray solid bars) and with a disability (yellow solid bars) and the female participants with no disability (gray striped bars) and with a disability (yellow striped bars) for the reaction time (RT) measures in (μsec; **C,D**) as a function of the Flanker Task in a controlled sound-attenuated environment. The data show a statistically significant effect of having a disability that reduced the % accuracy in males (*p* < 0.001***; **A**) and females (*p* < 0.001***; **B**) across all Flanker Test Conditions. Interestingly, neither males **(C)** nor females **(D)** exhibited any statistically significant effect of a disability that influenced RT across any of the Flanker Test Conditions. Data are shown as the Mean ± SEM, and bars without SEMs are because the value had little to no variability.

The results for males showed that the sensitivity of the eye gaze technology to detect any differences between students with and without disabilities for the *% accuracy measure* revealed a significant effect of *Disability* [*F*_(1)_ = 122.909, *p* = 0.001***, *η_p_*^2^ = 0.885] with a significant *pair-wise comparison* between the groups on all the *Flanker Test Conditions* (*p* = 0.001^###^). However, there was no significant effect of *Condition* [*F*_(3)_ = 1.805, *p* = 0.187 n/s], nor was there any significant *Disability X Condition interaction* [*F*_(1,3)_ = 1.805, *p* = 0.187 n/s] for the *% accuracy measure* (Figure 4A). The results for females showed that the sensitivity of the eye gaze technology to detect any differences between students with and without disabilities for the *% accuracy measure* revealed a significant effect of *Disability* [*F*_(1)_ = 84.145, *p* = 0.001***, *η_p_*^2^ = 0.397] and a significant effect of *Flanker Test Condition* [*F*_(3)_ = 0.2.647, *p* = 0.05*, *η_p_*^2^ = 0.058]. However, there were no significant *Disability X Flanker Test Condition interactions* [*F*_(1,3)_ = 0.774, *p* = 0.511 n/s] for the *% accuracy measure* (Figure 4B). The results for males showed that the sensitivity of the Flanker Task to detect any differences between students with and without disabilities for the *RT measure* revealed a trend approaching significance for an effect of *Disability* [*F*_(1)_ = 4.142, *p* = 0.059, *η_p_*^2^ = 0.206]. However, there was no significant effect of *Flanker Test Condition* [*F*_(3)_ = 0.092, *p* = 0.964 n/s], nor was there any significant *Disability X Flanker Test Condition interaction* [*F*_(1,3)_ = 0.074, *p* = 0.973 n/s] (Figure 4C). The results for females showed that the sensitivity of the Flanker Task to detect any differences between students with and without disabilities for the *RT measure* revealed a significant effect of *Disability* [*F*_(1)_ = 6.765, *p* = 0.01**, *η_p_*^2^ = 0.500]. However, there was no significant effect of *Flanker Test Condition* [*F*_(3)_ = 0.077, *p* = 0.972 n/s], nor was there any significant *Disability X Flanker Test Condition interactions* [*F*_(1, 3)_ = 0.219, *p* = 0.883 n/s] (Figure 4D). Overall, when examining the *Main Effect* of *Disability,* it was observed to be statistically significant for *% accuracy* [*F*_(1)_ = 73.441, *p* < 0.001***, *η_p_*^2^ = 0.338], and a *Gender X Disability Interaction* was also observed to be statistically significant for *RT* [*F*_(1, 1)_ = 11.158, *p* < 0.001***, *η_p_*^2^ = 0.072].

### 3.3 Experiment 3 findings

**Figure 5:**
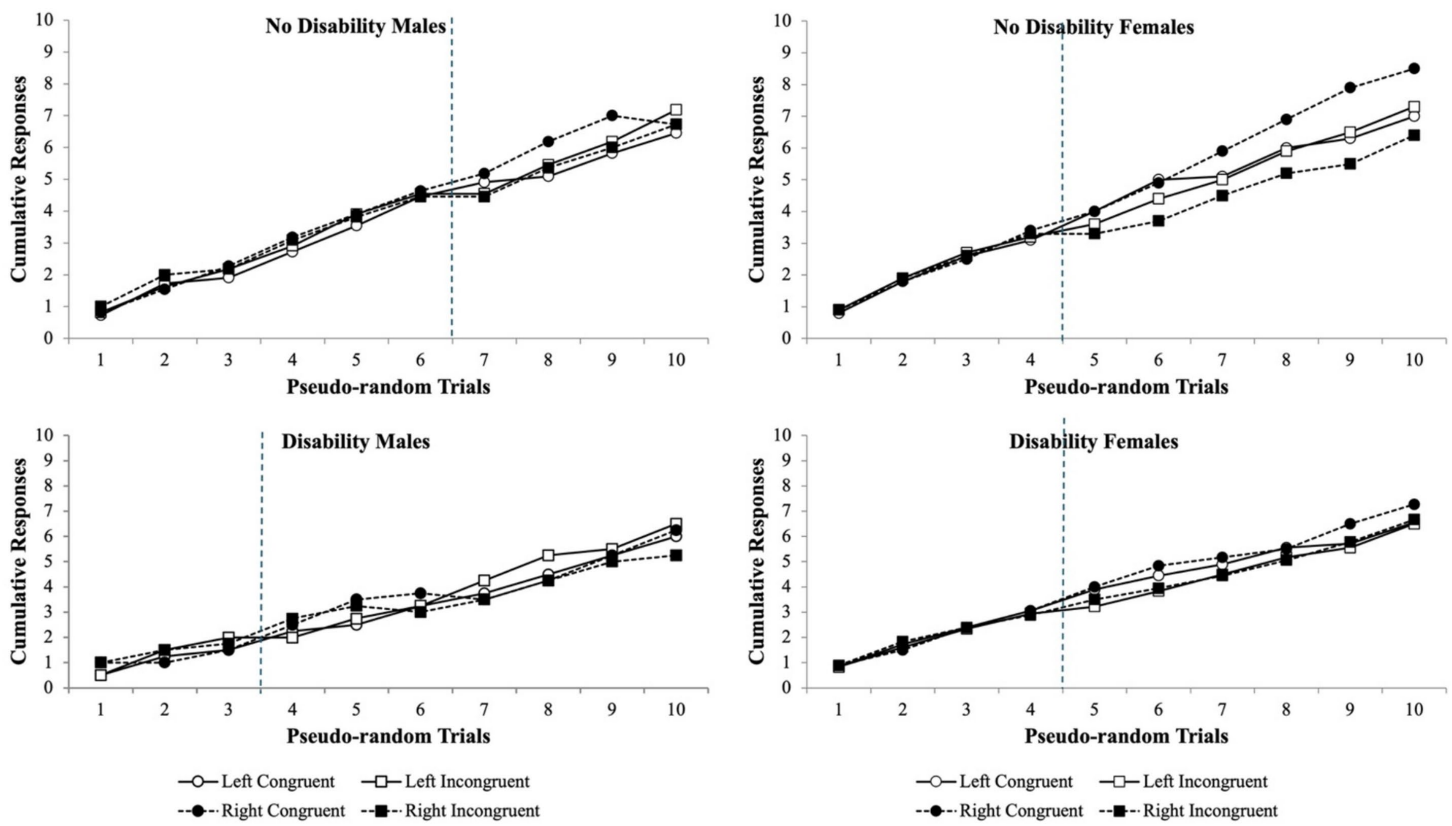
Pseudo-randomized trials from the Flanker Task experiment to illustrate how male participants with no disability (upper left panel) perform on the visual eye tracking assessment compared to male participants with a disability (lower left panel), female participants with no disability (upper right panel), and female participants without a disability (lower right panel) for the percent (%) accuracy of responses as a cumulative record. The data show the rate of improvement with trail-by-error experience from trail 1 to 10 for each of the Flanker test conditions. Over trials as a function of testing, some separation of the Flanker test conditions emerges (blue vertical dotted line used as threshold for this data separation) in which the variability in males with disabilities (lower left panel) is shifted leftward by 3 trials when compared to males with no disability (upper left panel), whereas in females the variability is similar. However, from trials 5 to 10 in females with no disability (upper right panel), there are more differences across Flanker test conditions than females with a disability (lower right panel). Data are illustrated as the means collapsed for each group for each test condition ± SEM, and markers without SEMs are because the value had little to no variability.

**Figure 6:**
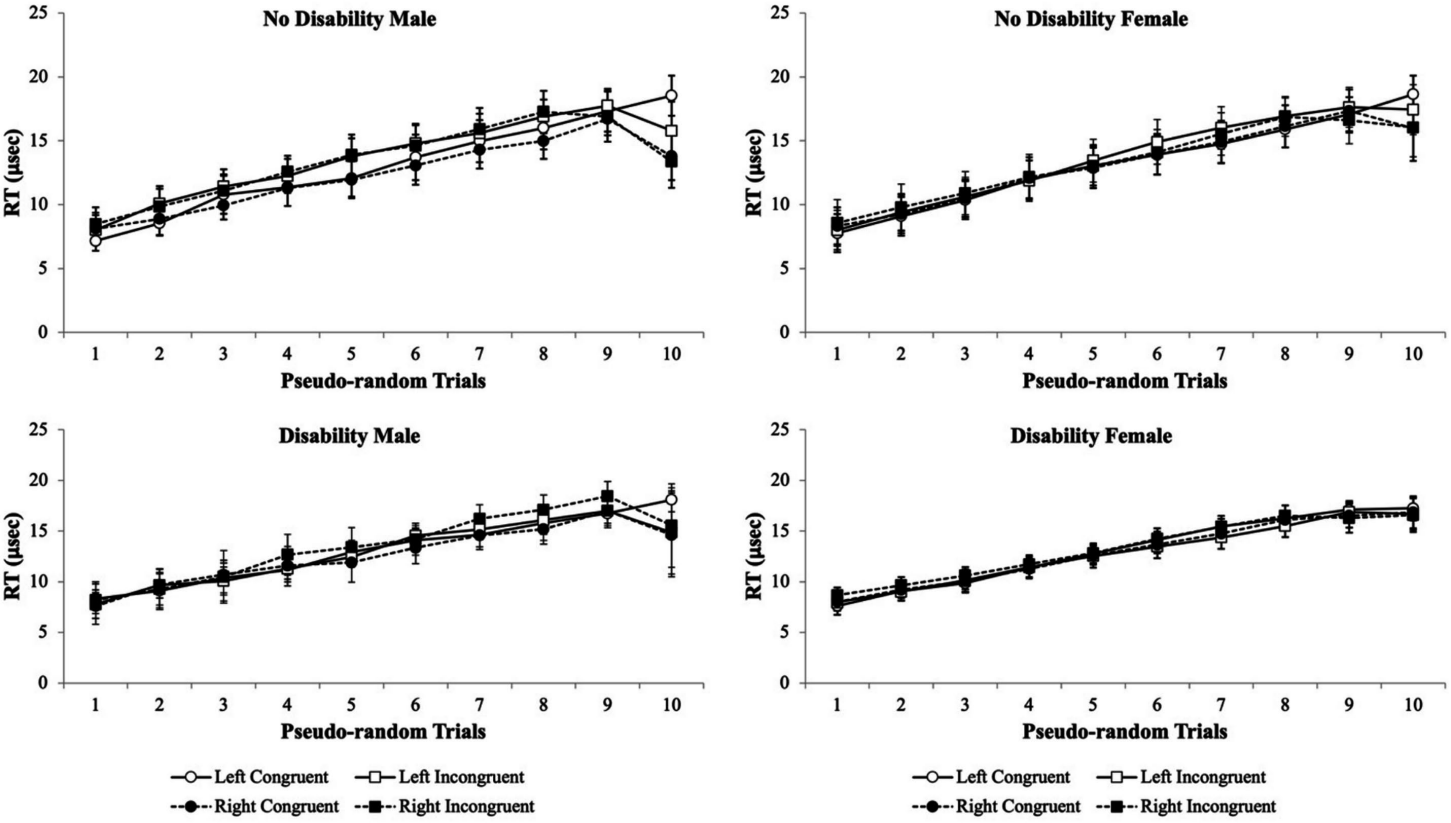
Pseudo-randomized trials from the Flanker Task experiment to illustrate how male participants with no disability (upper left panel) perform on the visual eye tracking assessment compared to male participants with a disability (lower left panel), female participants with no disability (upper right panel), and female participants without a disability (lower right panel) for the RT of responses as a cumulative record. The data show the rate of improvement with trail-by-error experience from trails 1 to 10 for each of the Flanker test conditions. Over trials as a function of testing, some separation of the Flanker test conditions emerges at the very last trial but is not different from any group. Data are illustrated as the means collapsed for each group for each test condition ± SEM, and markers without SEMs are because the value had little to no variability.

To assess and test the sensitivity of the Flanker Task to detect any differences between participants with and without disabilities in their ability to complete the Flanker task as before, the data were subjected to a pseudo-randomization of each test trial for each test condition. Thus, the change in test performance for each of the participants *% accuracy* is illustrated as cumulative records for males and females with and without a disability (Figure 5). The *% accuracy* cumulative record data show that the Flanker Task is sensitive in showing data separation as a function of trial as the participants advance through the test. Males without a disability typical showed a pattern of data separation at trials 6–7 (Figure 5; upper left panel), whereas males with a disability have a pattern of data separation that is shifted leftward at trials 3–4 for their *% accuracy* cumulative records (Figure 5; lower left panel). Interestingly, females without a disability (Figure 5; upper right panel) and with a disability (Figure 5; lower right panel) show similar data separation occurring at trials 4–5. However, the data separate more and become more variable for females without disabilities at trials 5–10 for their *% accuracy* cumulative records. With respect to the *RT* cumulative records, there were no differences in separation noted for males or females with and without disabilities and they began to show separation at trial 10 (Figure 6). Taken together, the Flanker Task was sensitive to detect shifts in males with disabilities when compared to males without disabilities and the lack of data separation in later trials with females with disabilities when compared to females without disabilities for *% accuracy* but not *RT* cumulative responses.

### 3.4 Experiment 4 findings

**Figure 7:**
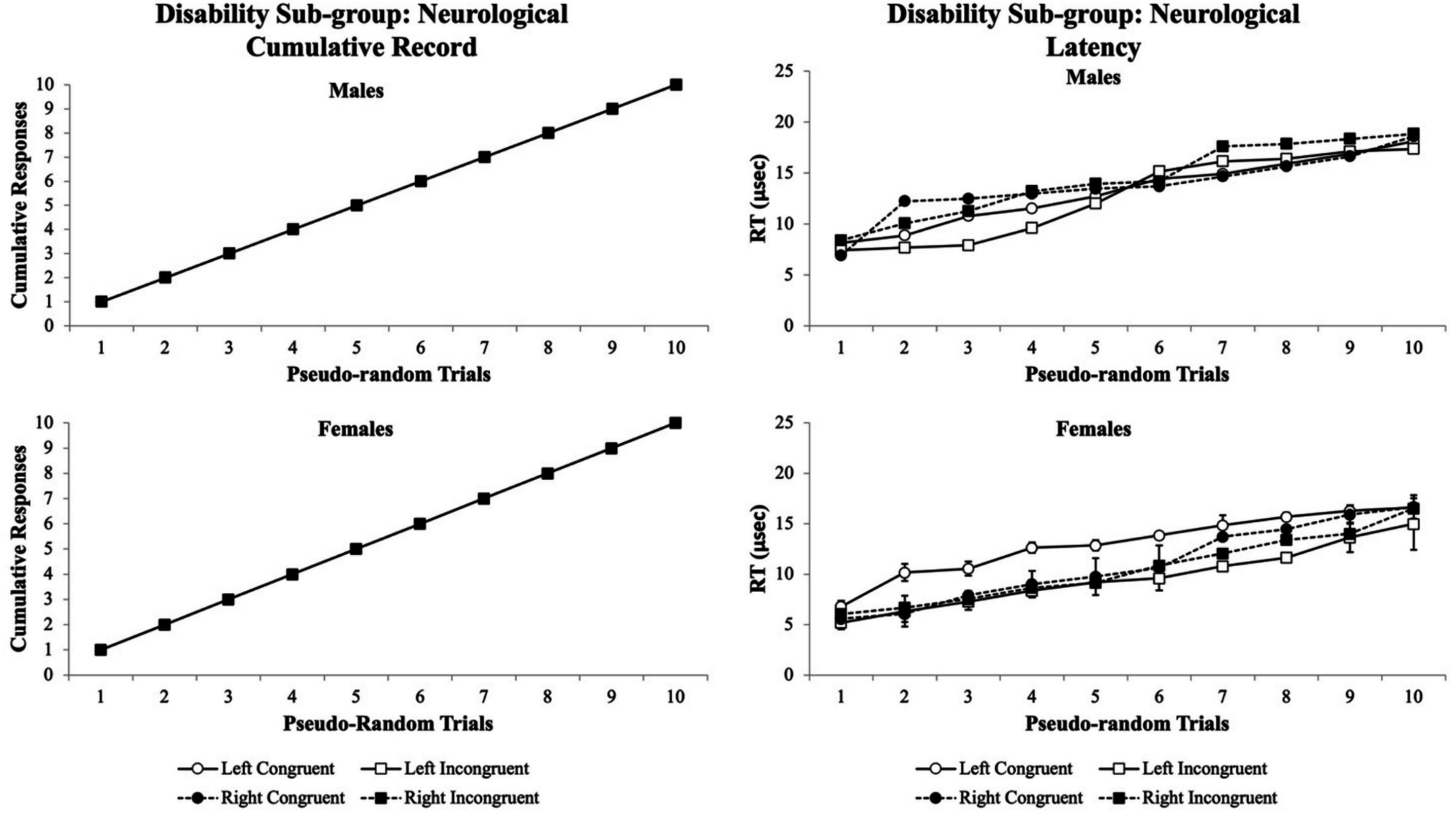
Male cumulative records % accuracy (upper left panel) and RT (upper right panel) and the female cumulative records % accuracy (lower left panel) and RT (lower right panel) for the neurological/health impairments sub-group. In males, variability was observed at trials 1–5, and females had more variability at trials 6–10. Notably, in females with neurological/health impairments, the males and females had nearly identical data patterns for the % accuracy cumulative records. The visual eye tracking system was sensitive for this group’s RT where the males showed more variability at trials 1–5 and the females showed more variability at trials 6–10. The females also had longer sustained RTs for the Left Congruent test condition throughout the experiment. Data are illustrated as the means collapsed for each group for each test condition ± SEM, and markers without SEMs are because the value had little to no variability.

To further assess how a specific type of disability may affect a person’s visual attention when conducting the Flanker Task, the triple-blinded data were disaggregated by type of disability while maintaining anonymity to assess how sensitive the technology would be. The following groups were broken down based upon disability category (i.e., ADD/AD/HD, learning/processing disabilities, emotional/psychiatric disorder, occupation/motor impairment, neurological/health impairment, and multiple disabilities). First, the visual attention patterns for the neurological/health impairment sub-group were examined. Figure 7 illustrates the visual attention patterns for males (upper panels) and females (lower panels) with neurological/health impairments. In this disability sub-group, the males and females performed nearly identical on their cumulative records for *% accuracy*. However, their *RT* cumulative records were different whereby males had more variability at trials 1–5 and females had more variability at trials 6–10. Notably, in females with neurological/health impairments, the Left Congruent condition had longer and sustained *RTs* throughout the experiment over other test conditions. This suggests that the visual attention performance for *RTs* could be used as a screening tool for students with neurological/health impairments.

**Figure 8:**
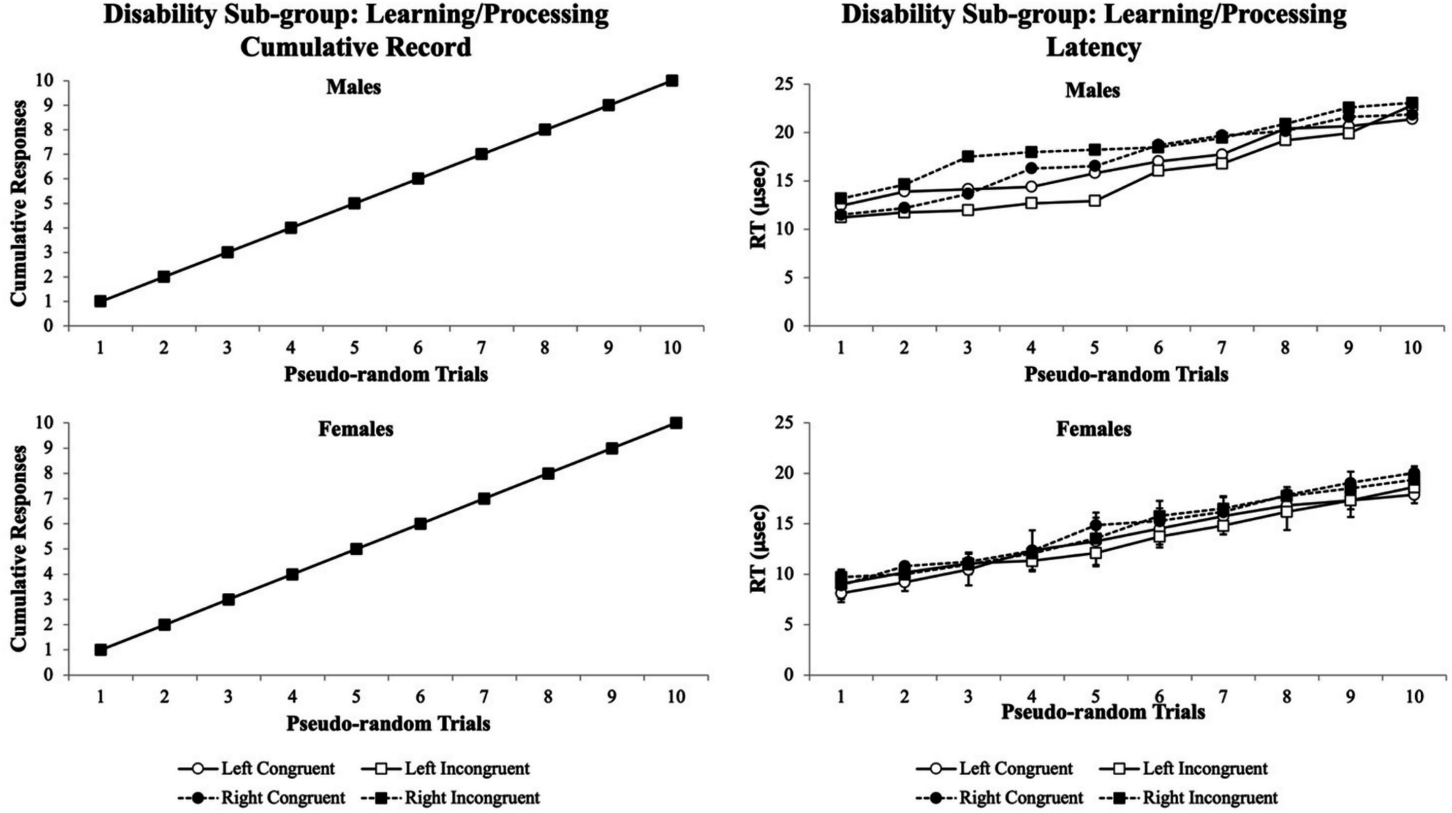
Male cumulative records % accuracy (upper left panel) and RT (upper right panel) and the female cumulative records % accuracy (lower left panel) and RT (lower right panel) for the learning/processing disorder sub-group. In males, variability was observed at trials 1–5, and females had more variability at trials 6–10. The males and females had nearly identical data patterns for the % accuracy cumulative records. The visual eye tracking system was sensitive for only male’s RT where the males showed more variability at trials 1–5. The females had stable patterns of RT cumulative records throughout the experiment, and the visual eye tracking data were insensitive to pick up any deviation in pattern. Data are illustrated as the means collapsed for each group for each test condition ± SEM, and markers without SEMs are because the value had little to no variability.

Second, the visual attentional patterns for the learning/processing disabilities sub-group were examined. Figure 8 illustrates the visual attentional patterns for males (upper panels) and females (lower panels) with learning/processing disabilities. In this learning/processing disabilities sub-group, the males and females performed nearly identical on their cumulative records for *% accuracy*. However, their *RT* cumulative records were different whereby only males showed more variability at trials 1–5 and females had stable *RTs* throughout the experiment over other test conditions. Notably, in females with learning/processing disabilities, the visual eye tracking data were insensitive to pick up any deviation in pattern. This suggests that the visual eye tracking system *RTs* cumulative records could be used as a screening tool for male students with learning/processing disabilities, but it might be difficult to determine whether that particular disability were the issue or if it were due to a neurological/health impairments as they shared the same pattern of *RT* cumulative records.

**Figure 9:**
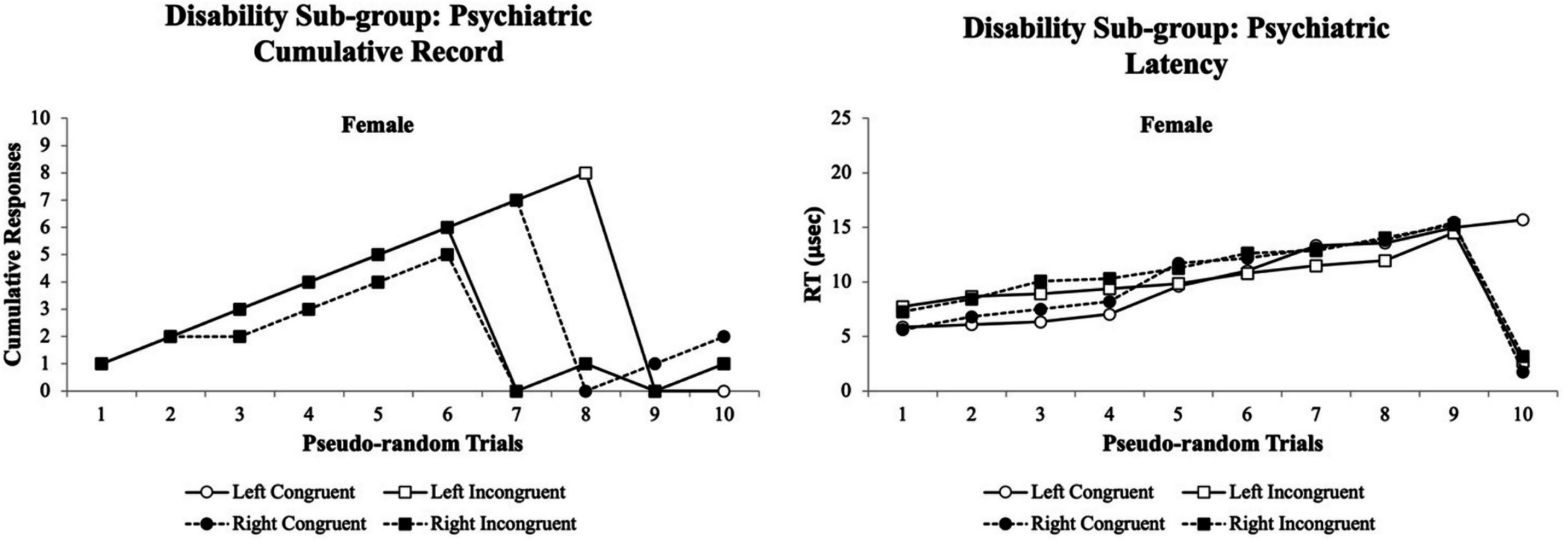
Female cumulative records % accuracy (left panel) and RT (right panel) for the emotional/psychiatric disorder sub-group. The females’ cumulative records % accuracy was highly variable at trials 6–10 with a steep decline in accuracy (left panel). In contrast, the females performed with a more flattened cumulative record for their RT (right panel) with very fast response times for the last trials of each test condition. The visual eye tracking system was sensitive for both female’s % accuracy and RT cumulative records throughout the experiment. Data are illustrated as the means collapsed for each group for each test condition ± SEM, and markers without SEMs are because the value had little to no variability.

Third, the visual attentional patterns for the emotional/psychiatric disorder sub-group were examined. Figure 9 illustrates the visual attentional patterns for females with emotional/psychiatric disorders. In this emotional/psychiatric disorder sub-group, only females were found to participate in the experiment. The females’ *% accuracy* cumulative records were highly variable at trials 6–10 with a steep decline in accuracy (left panel). In contrast, the females performed with a more flattened cumulative record for their *RT* (right panel) with very fast response times for the last trials of each test condition. This suggests that the visual eye tracking systems *% accuracy* and *RTs* cumulative records could be used as a screening tool for female students with emotional/psychiatric disorders. Since no males with emotional/psychiatric disorder participated in the study, it is unclear whether their visual eye tracking data would be similar to or different from the females with emotional/psychiatric disorders datasets.

**Figure 10:**
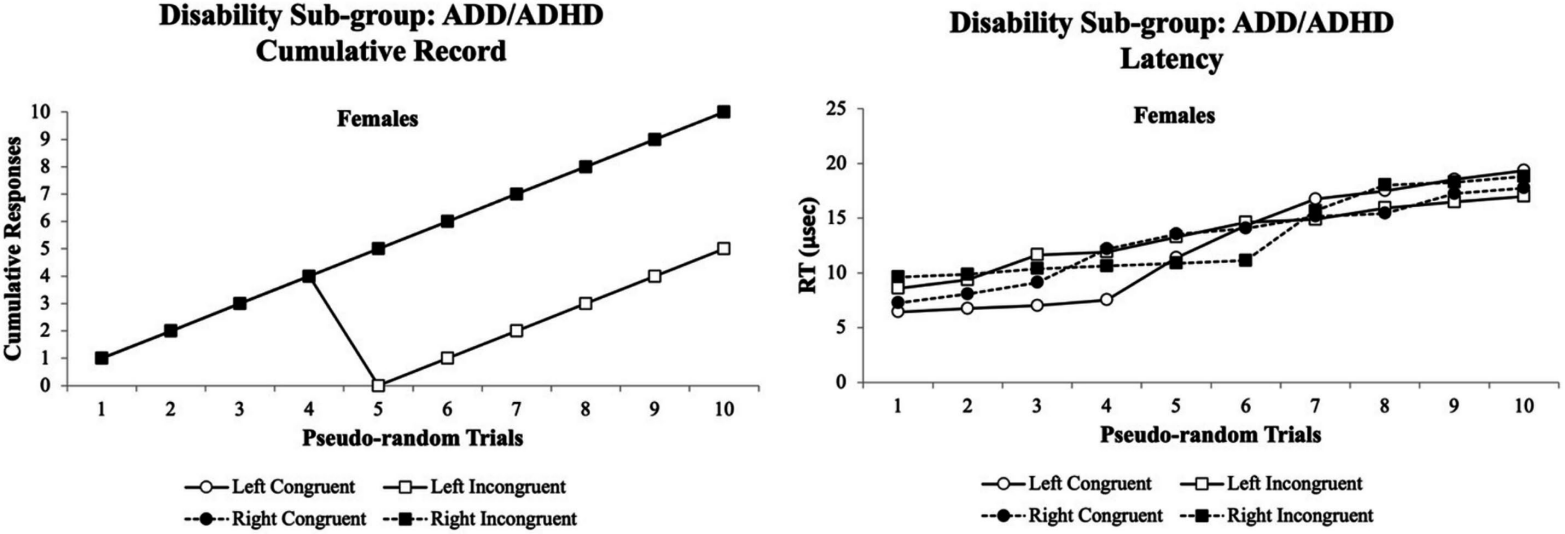
Female cumulative records % accuracy (left panel) and RT (right panel) for the ADD/AD/HD sub-group. The females’ cumulative records % accuracy was highly variable at trials 4–10 with a steep decline in accuracy and clear separation of Left Congruent and Left Incongruent trials with worse accuracy than the Right Congruent and Right Incongruent trials (left panel). In contrast, the females had a variable and more flattened cumulative record for their RT (right panel). The visual eye tracking system was sensitive for both female’s % accuracy and RT cumulative records throughout the experiment. Data are illustrated as the means collapsed for each group for each test condition ± SEM, and markers without SEMs are because the value had little to no variability.

Fourth, the visual attentional patterns for the ADD/AD/HD sub-group were examined. Figure 10 illustrates the visual eye tracking patterns for females with ADD/AD/HD. In the ADD/AD/HD sub-group, only females were found to participate in the experiment. The females’ *% accuracy* cumulative records were highly variable at trials 4–10 with a steep decline in accuracy and clear separation of Left Congruent and Left Incongruent trials with worse accuracy than the Right Congruent and Right Incongruent trials (left panel). In contrast, the females performed with a more variable and flattened cumulative record for their *RT* (right panel). This suggests that the visual eye tracking systems *% accuracy* and *RTs* cumulative records could be used as a screening tool for female students with ADD/AD/HD. Since no males with ADD/AD/HD participated in the study, it is unclear whether their visual eye tracking data would be similar to or different from the females with ADD/AD/HD datasets.

**Figure 11:**
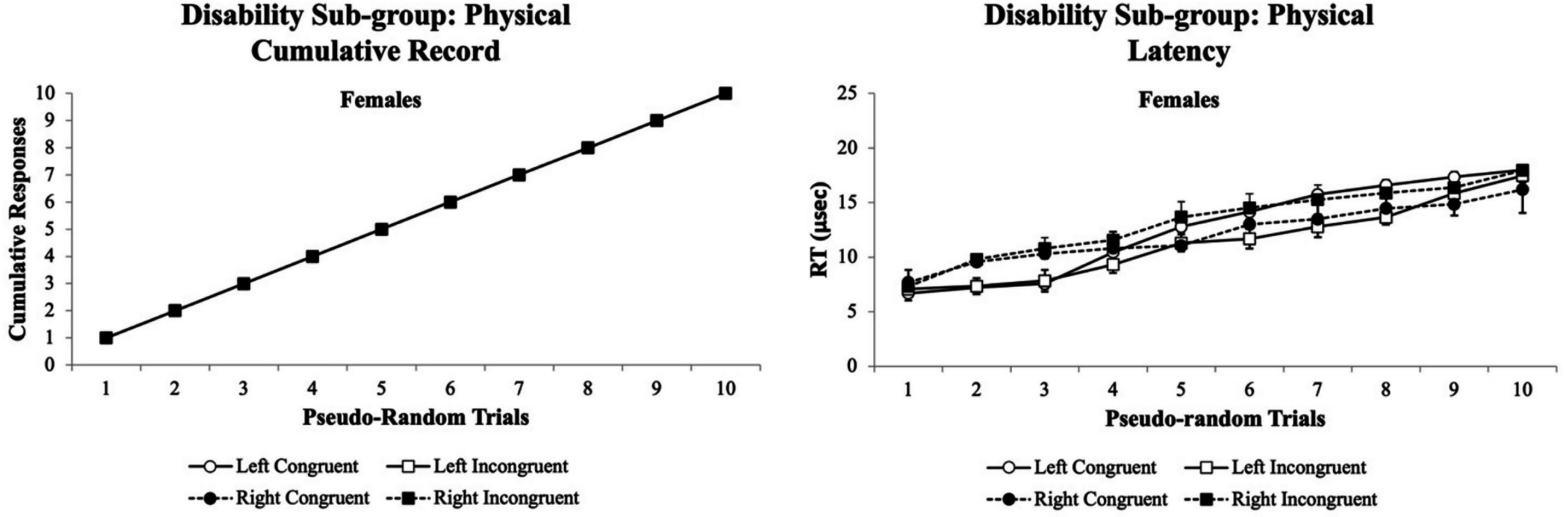
Female cumulative records % accuracy (left panel) and RT (right panel) for the physical occupation/motor impairment disorder sub-group. The females’ cumulative records % accuracy was absent of any variability across all trials (left panel). In contrast, the females had minor variability with a more flattened cumulative record for their RT (right panel). The visual eye tracking system was insensitive for both female’s % accuracy and RT cumulative records throughout the experiment. Data are illustrated as the means collapsed for each group for each test condition ± SEM, and markers without SEMs are because the value had little to no variability.

Fifth, the visual attentional patterns for the physical occupation/motor impairment disorder sub-group were examined. Figure 11 illustrates the visual attentional patterns for females with physical occupation/motor impairment disorders. In this disability sub-group, only females were found to participate in the experiment. The females’ cumulative records *% accuracy* was absent of any variability across all trials (left panel). In contrast, the females had minor variability with a more flattened cumulative record for their *RT* (right panel). The visual eye tracking system was insensitive for both female’s *% accuracy* and *RT* cumulative records throughout the experiment.

**Figure 12:**
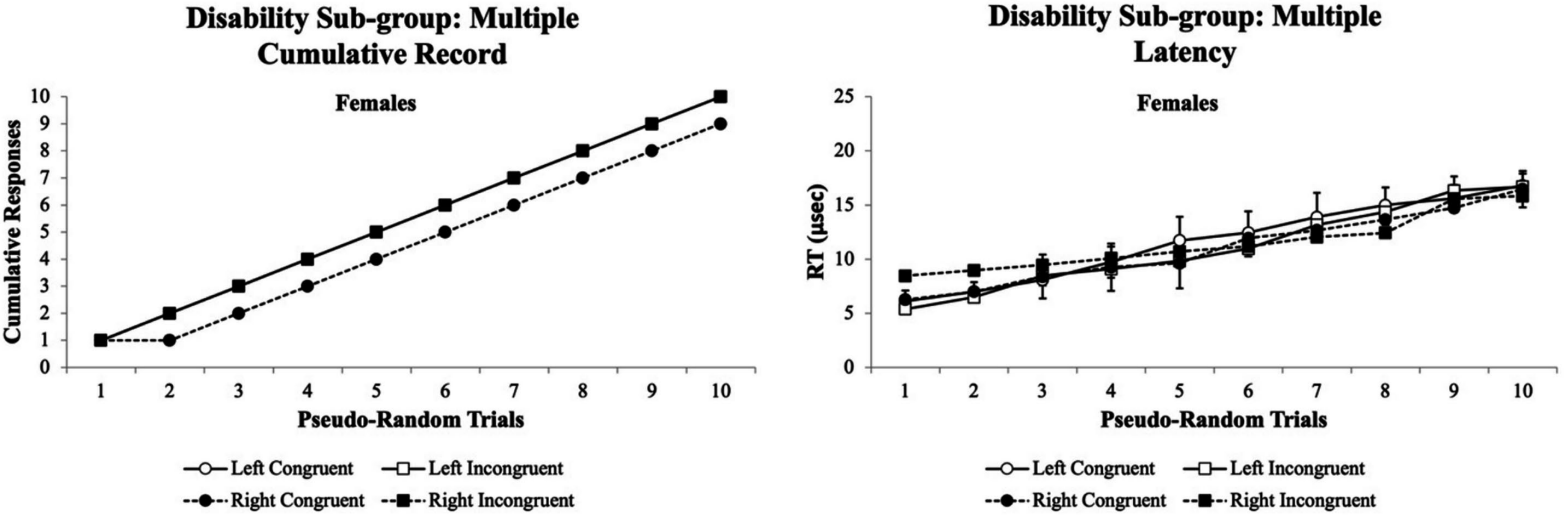
Female cumulative records % accuracy (left panel) and RT (right panel) for the multiple disability sub-group. The females’ cumulative records % accuracy showed a parallel pattern that separated the Congruent from the Incongruent test conditions at trials 2–10 that remained across all trials (left panel). In contrast, the females had minor variability with a more flattened cumulative record for their RT (right panel). The visual eye tracking system was sensitive for only female’s % accuracy and not their RT cumulative records throughout the experiment. Data are illustrated as the means collapsed for each group for each test condition ± SEM, and markers without SEMs are because the value had little to no variability.

Sixth, the visual attention patterns for the multiple disability sub-group were examined. Figure 12 illustrates the visual attention tracking patterns for females with multiple disabilities. In the multiple disability sub-group, only females were found to participate in the experiment. The females’ cumulative records *% accuracy* showed a parallel pattern that separated the Congruent from the Incongruent test conditions at trials 2–10 that remained across all trials (left panel). In contrast, the females had minor variability with a more flattened cumulative record for their *RT* (right panel). The visual eye tracking system was sensitive for only female’s *% accuracy and* not their *RT* cumulative records throughout the experiment.

## 4 Discussion

The pilot study sought to assess how visual attention and eye tracking combined with the Flanker Task could be used to assess students visually guided working memory and fronto-executive functions as cognitive processes, using *% accuracy* and *RT* response measures, and furthermore, if those types of measures could be used as a prognostic tool to determine which students would be able to benefit from visual screen-related accommodative software. To provide more context of the visual screen-related accommodative software, the present study sought to consider the following: (1) Kurzweil 3,000; (2) ZoomText; (3) Fusion; and (4) Onyx. Kurzweil 3,000—a sophisticated text-to-speech software that integrates auditory and visual learning. The software allowed users to choose from 100 s of different voices, accents, dialects, and speeds to have the text read to them, while on screen, color blocks or lines can chunk the visual text by paragraph, line, or word for word depending on the user’s level of visual acuity and attention. Users can click on any text for a definition if they cannot identify it either visually or aurally. Kurzweil 3,000 can be applied to any digital learning content and is the most widely prescribed and most popular assistive technology for our students with visual disabilities. ZoomText—an integrated screen magnification and clarification software that spans 1x-6x and can read aloud selected text or what the user is typing. Fusion—a highly sophisticated integration of ZoomText and JAWS reader software. Fusion makes the combination of magnification and screen reader seamless. JAWS reading capabilities are natural and expressive, and it moves with ease as a user navigates complicated document magnifications. Onyx—a combination video monitor and magnifier that can be used for both portable objects and distance viewing. Users can view smart boards, screens, and chalkboards on their personal video monitor up to 133x and as far as across an auditorium. Users can display the visuals in a wide variety of colors and gray scales, as well as reading lines, shades, and screens to suit their visual needs. Users can also lock in a moving image to scrutinize details at their own pace.

The first experiment demonstrated methodological internal negative controls for the environment whereby when students without disabilities were tested in a noisy environment, their *% accuracy* decreased but not their *RT* (Figures 3A–D). This suggested that visual attentional mechanisms directed toward their decision-making processes were subject to interference that negatively influenced their working memory and fronto-executive functions in the Flanker Task resulting in decreased % accuracy (Figures 3A,B). Notably, these same environmental distractions did not affect their latency by which the participants cognitively selected their response and engaged in the behavioral action to select their choice response key (Figure 3C,D). This suggested that the noisy environment was sensitive enough to cause cognitive interference with the visual attentional demands of the Flanker Task and that it could be captured with the methods deployed herein. The gender differences that were noted between the noisy and controlled environments showed that females were more distracted than males in this noisy environmental condition.

Next, the second experiment was conducted within a controlled Neuropsychology Laboratory sound-attenuated setting to maximize participant concentration and limit distractibility showed that students with disabilities (i.e., as an aggregated group) exhibited a decreased *% accuracy* in their visual attention for both males and females with no gender differences observed (Figures 4A,B). Moreover, regardless of the type of disability from the aggregated group, it did not affect their *RT* by which the participants cognitively directed their behavioral actions to select their choice response key (Figures 4C,D). This suggested that the Flanker Task was sensitive enough to capture the aggregated students with disabilities’ visual attentional differences in their responding to the Flanker Task. This finding provided confirmation that if students with disabilities were to have visual difficulty with searching and looking for target stimuli on screen that they may not be well suited for accommodative technologies that either requires them to look at distinct areas of the computer screen to select items or move as a cursor. Instead, these students may benefit from alternative accommodative technologies or technologies that could include such visual search needs (i.e., Kurzweil 3,000, ZoomText, Fusion, and/or Onyx). Furthermore, the Flanker Task could be used as a simple screening tool for such purposes to determine which students may or may not have visual attentional issues.

Subsequently, the third experiment maintained the aggregated disability group and compared their cumulative records for both the *% accuracy* and *RT* to the group without disabilities. The cumulative records allowed for a more scrutinized approach to evaluating at what point across the 10 trials did the group with disabilities visual attention deviate from the group without disabilities. For males, this deviation point began at trial 6 and continued variability through trial 10, whereas in females this observation was shifted leftward and began at trial 4 and continued variability through trial 10 (Figure 5). Interestingly, for the *RTs,* the male’s deviation point began at trial 3 with much less variability through trial 10, whereas for the female’s deviation point it was shifted slightly rightward and began at trial 4 with much less variability through trial 10 (Figure 6). These data revealed that the Flanker Task is sensitive in screening for visual attention differences in students with and without disabilities with gender-specific shifts for *% accuracy* and *RTs*.

Finally, the fourth experiment disaggregated the disability group into six sub-groups to determine whether a finer analysis could reveal specific visual attention behavioral phenotypes to be used as a prognostic screening tool for matching students with appropriate accommodative technologies (i.e., Kurzweil 3,000, ZoomText, Fusion, and/or Onyx). For the neurological health/impaired sub-group, for both males and females, the cumulative records for the *% accuracy* were nearly identical with a typical behavioral pattern. However, the *RTs* showed more sensitivity to the Flanker task test conditions, suggesting that *RT* cumulative records in neurological/health impaired students could be screened with the Flanker Task (Figure 7). Similarly, the same data findings in the learning/processing disabilities sub-group matched the cumulative records *% accuracy* and *RTs* as the neurological/health impairments group (Figure 8). This poses two unique situations (1) that both neurological/health impairments and learning/processing disorder students could be screened using the cumulative records *RTs*, but (2) it would not be able to differentiate between these two sub-groups.

For the emotional/psychiatric disorder sub-group in which only females volunteered, they exhibited the most deviations in their cumulative records for both the *% accuracy* and the *RTs* which began at trial 6 and trial 9 with steep declines in performance, respectively (Figure 8). These data suggested that the Flanker Task was very sensitive in picking up these cumulative record differences in their visual attention performance of the emotional/psychiatric disorder sub-group as a screening tool. Interestingly, for the ADD/AD/HD sub-group in which only females volunteered, they exhibited a deviation in there *% accuracy* cumulative records that began at trial 4 and had a parallel visual attention performance with worse performance for left than right Flanker Task stimuli. For the *RT* cumulative records, the ADD/AD/HD sub-group had deviations that began at trial 1 and had continued variability through trial 10 (Figure 9). These data suggested that the Flanker Task was very sensitive in picking up these cumulative record differences in their visual attention performance of the ADD/AD/HD sub-group as a screening tool.

For the physical sub-group in which only females volunteered, they exhibited typical cumulative records for *% accuracy*, but their *RT* cumulative records showed a similar pattern as the ADD/AD/HD sub-group with slightly less variability (Figure 10). Interestingly for the multiple disability sub-group in which only females volunteered, they exhibited a parallel cumulative record for *% accuracy* with worse performance on the congruent than incongruent stimuli. Furthermore, their *RT* cumulative records were similar to the ADD/AD/HD and emotional/psychiatric disorder sub-groups (Figure 11). These data suggested that the Flanker Task was very sensitive in picking up the *% accuracy* cumulative record differences in their visual attention performance of the multiple disability sub-group. Finally, the occupation/motor impairment sub-group exhibited similar *% accuracy* and *RT* cumulative records as the learning/processing disabilities sub-groups (Figure 12).

In summarizing the findings, the present pilot study’s methods showed preliminary data that the Flanker Task could detect visual attentional differences in the pattern of the *% accuracy*, *RTs,* and the cumulative records for both *% accuracy* and *RTs* of students with and without disabilities. Furthermore, when students with disabilities were disaggregated using the triple-blind procedure, the Flanker Task was able to detect different patterns in their visual attentional performance that could be used as a quick 10-min screening tool to best match students with specific visual accommodative technologies to help them complete their psychology major (i.e., Kurzweil 3,000, ZoomText, Fusion, and/or Onyx).

### 4.1 Assessing the Flanker Task for prescriptive visual accommodative technologies

In reviewing the literature, there are scant articles utilizing the Flanker Task in college-aged students with varying disabilities to determine whether it could be used as a prescriptive tool to assess visual accommodative needs. First, it is important to note that when implementing a Flanker Task, that researchers testing participants with and without disabilities are to ensure the congruent and incongruent trials are presented with equal frequency, to reduce the likelihood of an increased Flanker effect (Lehle and Hübner, 2008), and to be mindful of location stimulus selectivity as another by-product threatening internal validity (Lehle and Hübner, 2008). As failure to do so would limit interpretations of any Flanker Task outcomes on either population of college students. The present pilot study was able to parse populations of college students with varying disabilities and determined that it could be an effective tool in selecting certain populations of students with disabilities that might benefit from visual accommodative technologies. Prior work on large sample of children (*N* = 272) with and without AD/HD has reported that when presented with the Flanker Task and the Simon Task, they exhibited performance deficits in RT, percentage of errors, and efficiency in detecting incongruent trials when compared to control trials, thereby suggesting increased problems with working through interference test conditions (Mullane et al., 2009). However, studies on college-aged adults with AD/HD replicating this study have yet to be done to see whether this reduced interference skillset is recovered or exacerbated with age, and whether early interventions can serve as a mitigating intervening variable. In adults without disabilities, fronto-executive control can be correlated in the Flanker Task to an N200 event-related potential (ERP) (Kopp et al., 1996), but whether this same ERP would occur at the same time in individuals with disabilities and how it might shift either leftward or rightward relative to individuals without disabilities remains to be elucidated. A study in college-aged students in Cyprus University of Technology attempted to pilot correlates of EEGs and eye tracking and found that the eye tracking data correlated with different cognitive abilities in individuals without disabilities (Nisiforou and Laghos, 2016). Thus, if differing cognitive states could be differentiated in relation to eye tracking in individuals without disabilities, then logically it would be within reason to assume that further differentiation in these correlates may uniquely present with individuals of varying disabilities. Despite the gaps in the literature, the present pilot study proved promising as a next step to systematically begin to address this gap in the literature. What is also promising is that prior reports have shown that altered self-regulatory control of an individual’s own eye movements can be a key factor related to neurodegenerative movement disorders (Gorges et al., 2014) and could also be perhaps prescriptive in this same context. It is important to note that combining EEG or ERP with eye tracking may be the best approach to determine the cognitive strategy the person is using when they are attending to specific stimuli. Cooke (2006) aptly noted that relying on eye tracking alone that researchers can understand where the participants are looking but not “why”? It is through this framework that the present pilot study is suggesting using another correlate to overcome the “why” problem. If students can be grouped by a disability category as done herein and under these assumptions, which have their own limitations, that the individuals in a specific group would have its own distribution of their cognitive states, then we could perhaps infer from their eye tracking pattern and behavioral responses in the Flanker Task would correlate with their disability type. If future studies can repeat this work, build additional datasets that match using the same disability categories, then more aggregated data across studies, meta-analyses, and the like could be done to validate both small population samples to screen and larger population samples to run more complete studies. As most colleges are seeing an increase in enrollment of students with a range of disabilities having the ability to screen them with an eye-tracker and a simple Flanker Task in less than 20 min to determine whether they would benefit from a visual accommodative technology may have a critical positive impact on their education. If they were to benefit greatly from the visual accommodative technologies available at their college, they may find a new set of supports for them facilitating their persistence and timeliness to degree completion. However, this would require students to self-disclose to receive such support services in college, and there are many students that may not self-disclose making such prescriptive screening efforts self-limiting. Notably, if a greater number of students with disabilities were to benefit from such a prescriptive screening process, data collected, publicly shared on its usefulness, and student impacts, then perhaps more students with disabilities may come forward, self-disclose, and seek such supports and services in college. Thus, more work is warranted in exploring these factors. This pilot dataset will hopefully influence institutional policy changes to improve visual accommodative technologies that will match the needs of undergraduate students with disabilities to provide them with the resources they need to complete their psychology degrees in an optimal manner. Finally, this pilot study may become the beginning of a richer conversation on screening and assessment methods for undergraduate college students with disabilities to help them in selecting the best matched accommodative technologies to support them as early college interventions for adult learners.

### 4.2 Study limitations

The present pilot study is not without limitations. First, the sample size for the students with disabilities was quite small and the overall pool lacked equal representation for males and females. There can be several reasons for this and as such make it difficult for any comprehensive research study; hence, why the present pilot study is being used to start an important societal conversation regarding the matter. Students with disabilities may feel less capable and confident to enroll into college and may become less certain of the kinds of supports, assistance, and services that can be provided to them at the college level, which may make them less likely to enroll in college. In contrast, even if they enroll in college, as an adult is it their choice to self-identify as having a disability which is very different from their earlier ages in which their parents will lead such discussions. It is very possible that as students with disabilities that have successfully achieved the educational ability to enroll in college, may seek to reduce their stigma and labels to integrate more into the college experience without being detected as having a disability. They can cause two problems: (1) less participants with disabilities for studies such as the present one conducted and (2) students with disabilities that are unwilling to self-identify may then alter the students without disability data when aggregated together thereby counteracting such studies to be as clean as possible in their data findings. To this point, it may not be entirely possible to conduct full statistically sound sample sizes for such study purposes and may make it prudent for a series of pilot studies such as the present one to be published first. Then, perhaps a meta-analysis of these varied pilot studies could be used to attempt to resolve this issue, or retrospective analysis may also prove useful in overcoming the problem. It may take many years for the required comfortability and trust to take place in college students with disabilities to self-identify to be studied in an effort to help other students with similar and different disabilities at the college level. The unwillingness to self-identify as well as a fear of stigmatization is a reasonable attribution for the relatively small size of the pool of students with disabilities that were examined in the present study that corroborate with prior reports (Grimes et al., 2017). Furthermore, by National Science Foundation, National Center for Science and Engineering Statistics (2018) estimates, 78% of all psychology undergraduates are women, and this is consistent with the 9:1 ratio of females to males in this present study, since the participant pool was primarily comprised of psychology majors. It should be noted here that while there is a small gender difference between students with identified disabilities, approximately 64% of students with disabilities are female, as compared with 57% of students without disabilities who are female (U.S. Government Accountability Office, 2024), and this difference was washed out given the enormous gender differences in the population of students majoring in psychology. These two very real challenges have limited these findings to a pilot study. Future research, with a larger participant pool of undergraduate college participants with disabilities, should explore the data trends as indicated by this pilot study. This would permit future researchers to determine whether the six disability sub-groups exhibit different *patterns* of responses with respect to the visual attentional data and the visual eye tracking heat maps, as well the degree to which to those patterns can be used to characterize the response patterns across groups.

### 4.3 Future outlook

This pilot study was the first of its kind to evaluate college-age students at the undergraduate level and to see whether using cognitive psychology tests such as the Flanker Task could be used to detect differences in visual attention and eye-gaze patterns. Unfortunately, much of the literature on individuals with disabilities are limited to children and adolescents and there are less reports on adults with disabilities in college. Moreover, the literature is scant on examining differences in visual processes of students with disabilities, and perhaps it is due to the lack of clarity in grouping students in ways that may make it easier to identify similarities and differences in their abilities despite their disabilities. Much of these codification systems have evolved substantially over the years, and this has offered a more wide-spread understanding of the range of disability for a given condition as well as the increased inclusivity of people along a neurodiversity spectrum. The students with disabilities in the present study were codified into six main categories: learning disabilities (LD), emotional or psychiatric conditions (EPC), orthopedic or mobility impairments (OMI), attention-deficit/hyperactivity disorder (AD/HD), health impairments (HIs), and multiple disabilities (MDs). These groups proved to be useful when examining them for eye tracking and visual attention tests using the Flanker Task. What can be learned from this pilot study is that future studies that want to build upon the present work could attempt to replicate what was done herein to see whether the same findings arise or with larger sample sizes could build upon our population datasets (i.e., using statistical Jack Knifing, Boot Strapping, or Monte Carlo methods) to simulate and build upon the sample size to see how the data might look like if it approached a large sample simulating a normal distribution. However, cation must be exercised as mathematical modeling may serve to provide insight and direction but may not always accurately model how humans might respond to certain stimuli or conditions. Thus, the best approach would be to recruit more students with disabilities to participate in similar kinds of study in the future. This recruitment effort depends on a balance of very sensitive issues regarding student confidentiality, triple-blinded processes, collaboration with the college’s students with disability services department, and the willingness for students with disabilities to participate in the study. These factors are all predicated on whether students with disabilities enroll into college and self-identify, which can be a difficult challenge for many of the reasons discussed earlier. Thus, if the field wants to develop more supports for students with disabilities in colleges at the undergraduate level, perhaps more collaborative opportunities between psychological researchers, psychological teachers/educators, supports for students with disabilities, and the administration should develop strategic plans to afford a more neurodiverse population of students with new assessment tools to help them along their most formative educational years. The findings from the present study collectively show that the Flanker Task was sensitive to parse students with differing disabilities to be screened for visual or other disability accommodative software that can help them with their college studies (i.e., Kurzweil 3,000, ZoomText, Fusion, and/or Onyx). However, since this is a pilot study, more work will need to be done to replicate, approach more of a meaningful sample size, and help follow up with pre- and post-test analyses of using such accommodative software from these screening processes. This study also suggests that perhaps other classical cognitive tests, such as the Flanker Task, could also be used in a similar manner, but that remains to be elucidated. Notably, such efforts will take some time before we can truly screen and facilitate students with disabilities at the college level in the ways the present study began to explore.

## 5 Conclusion

The pilot study is a promising demonstration that a 10-min Flanker Task can be used as an effective screening tool to match students with disabilities with appropriate accommodative technologies based on their visual attentional abilities. While certainly there is need for deeper investigation, with larger sample sizes of students with various types of disabilities and more equal male and female representation, to fully explore these differences in these types of Flanker Tasks, this pilot study demonstrates that relatively simple and minimally cost-effective tools can enable often under-resourced colleges and universities to address the needs students with different types of disabilities face and to provide them appropriate types of accommodations and support (i.e., Kurzweil 3,000, ZoomText, Fusion, and/or Onyx). A seemingly peripheral and yet very important additional benefit is that the exploration of these kinds of tools and strategic approaches may also provide opportunities for hands-on research experiences for undergraduate students in the field of cognitive neuroscience that include students with disabilities. These types of opportunities are often lacking in PUI institutions serving diverse populations, especially when the diversity term is used to include neurodiverse populations as well as racial and ethnic diversity. Future studies will seek to address questions that this pilot has opened. Until then, what can be stated is that the Flanker Task may be an underexplored resource for colleges and universities to consider for the purposes described in this study.

## Data Availability

The original contributions presented in the study are included in the article/supplementary material, further inquiries can be directed to the corresponding author.
